# Oscillation and asymptotic properties of a class of second-order Emden–Fowler neutral differential equations

**DOI:** 10.1186/s40064-016-3622-2

**Published:** 2016-11-10

**Authors:** Rui Wang, Qiqiang Li

**Affiliations:** School of Control Science and Engineering, Shandong University, Jinan, 250061 Shandong People’s Republic of China

**Keywords:** Oscillation, Second-order differential equation, Emden–Fowler equation, Nonpositive neutral coefficient, 34K11

## Abstract

We consider a class of second-order Emden–Fowler equations with positive and nonpositve neutral coefficients. By using the Riccati transformation and inequalities, several oscillation and asymptotic results are established. Some examples are given to illustrate the main results.

## Background

In this paper, we study a second-order delay differential equation

1 $$\left( r(t)|z^{\prime }(t)|^{\alpha -1}z^{\prime }(t)\right) ^{\prime }+f(t,x(\sigma (t)))=0,\quad t\ge t_0$$

where $z(t)=x(t)+ p(t)x(\tau (t))$ and $\alpha$ is a positive constant. Throughout this paper, we assume that

- ($$H_{1}$$) $$r,\;p\in {\mathrm {C}}([t_0, \infty ),{\mathbb {R}}),\; r(t)>0,\; 0 \le p(t)\le 1,\;\text {and}\; \int _{t_0}^{\infty }r^{-1/\alpha }(t) {\mathrm{d}}t=\infty ;$$
- ($H_{2}$) $r,\;p\in {\mathrm {C}}([t_0, \infty ),{\mathbb {R}}),\; r(t)>0,\; -1< -p_0 \le p(t)\le 0,\;\text {and}\; \int _{t_0}^{\infty }r^{-1/\alpha }(t) {\mathrm{d}}t=\infty ,$ where $p_0$ is a positive constant;
- ($$H_{3}$$) $$\tau \in {\mathrm {C}}([t_0, \infty ),{\mathbb {R}}), \; \tau (t)\le t, \; \text {and} \; \lim _{t\rightarrow \infty }\tau (t)=\infty ;$$
- ($$H_{4}$$)$$\sigma \in {\mathrm{C}}^{1}([t_0, \infty ),{\mathbb {R}}),\; \sigma (t)\le t,\; \sigma^{\prime}(t)>0,\;\text {and} \; \lim _{t\rightarrow \infty }\sigma (t)=\infty .$$
- ($H_{5}$) $f\in {\mathrm {C}}([t_0,\infty )\times {\mathbb {R}},{\mathbb {R}}),\;uf(u)\ge 0\; \text {for all}\; u\ne 0,$ and there exist a positive constant $\beta \;$ and a function $\; q(t)\in {\mathrm {C}}([t_0,\infty ),(0,\infty ))\; \text {such that}\; (f(t,u)/u^{\beta })\ge q(t),$ for all $u\ne 0$, where $1<\beta \le \alpha .$

It is recognized that Emden–Fowler equations have a number of applications in physics and engineering; see, e.g., Berkovich (1997). As a result, there has been a great deal of interest in investigating the oscillation or nonoscillation of differential equations; see, e.g., Hale (1977), Džurina and Stavroulakis (2003), Li (2004), Li et al. (2011, 2013, 2015), Li and Rogovchenko (2015), Erbe et al. (2009), Manojlović (1999), Wang and Yang (2004), Wang (2001), Liu et al. (2012), Shi et al. (2016), Baculíková and Džurina (2011), Bohner and Li (2014), Yang et al. (2006), Xu and Meng (2006, 2007). As we known, many results of half-linear or nonlinear equations with positive neutral coefficients were established, see, e.g., Baculíková and Džurina (2011), Erbe et al. (2009), Li et al. (2011, 2013), Li and Rogovchenko (2015), Liu et al. (2012), Shi et al. (2016), Yang et al. (2006), Xu and Meng (2006, 2007). The equations with nonpositive neutral coefficients have been applied to practical life; see, for instance, Brayton (1966) and Kuang (1993, sec. 1.1.7) provided a model about the system with lossless transmission lines. And, there have been a few oscillation and asymptotic results of the equations with nonpositive neutral coefficients, see, e.g., Bohner and Li (2014), Erbe et al. (2009), Li et al. (2015), Yang et al. (2006). In the following, we provide some background details which motivated our research. Manojlović (1999), Wang (2001), and Wang and Yang (2004) considered the half-linear differential equation

2 $$\begin{aligned} \left( r(t)|x^{\prime }(t)|^{\alpha -1}x^{\prime }(t)\right) ^{\prime }+q(t)|x(t)|^{\alpha -1}x(t)=0, \end{aligned}$$

and gave some different oscillation results by using an inequality due to Hardy, Littlewood and Ploya and averaging functions. Motivated by these ideas, many scholars extended the results to delay differential equations or neutral delay differential equations. Džurina and Stavroulakis (2003) and Li (2004) expanded the Eq. (2) to the delay differential equation

$$\begin{aligned} \left( r(t)|x^{\prime }(t)|^{\alpha -1}x^{\prime }(t)\right) ^{\prime }+q(t)|x(\tau (t))|^{\alpha -1}x(\tau (t))=0. \end{aligned}$$

Xu and Meng (2006, 2007) extended (2) to the neutral delay differential equation

3 $$\begin{aligned} \left( r(t)\left( \left[ x(t)+ p(t)x(\tau (t))\right] ^{\prime }\right) ^{\alpha }\right) ^{\prime }+q(t)(x(\sigma (t)))^{\alpha }=0 \end{aligned}$$

provided that $0\le p(t)\le 1$. Li et al. (2015) established some oscillation and asymptotic results to (1) in the case where $-1<p(t)\le 0$. By using Riccati transformation, Erbe et al. (2009) proposed some oscillation and asymptotic results for (1), under the assumptions that $0\le p(t)<1$ and $-1< p(t)< 0$. Liu et al. (2012) considered the the following equation

4 $$\begin{aligned} \left( r(t)\left| \left[ x(t)+ p(t)x(\tau (t))\right] ^{\prime }\right| ^{\alpha -1}z^{\prime }(t)\right) ^{\prime }+q(t)|x(\sigma (t))|^{\beta -1}x(\sigma (t))=0 \end{aligned}$$

in the case where $0\le p(t)\le 1$ and $\alpha \ge \beta >0$. They established some oscillation and asymptotic criteria by employing averaging technique and Riccati transformation. Shi et al. (2016) extended the results of Liu et al. (2012) to dynamic equations on time scales provided that $0\le p(t)\le 1$ and $p(t)> 1$.

However, the results of Liu et al. (2012) and Shi et al. (2016) cannot be applied to Eq. (1) due to $-1<p(t)\le 0$ in (1), but, in Liu et al. (2012), Shi et al. (2016) the assumption is $0\le p(t)\le 1$ or $p(t)>1$. Similarly, the results in Erbe et al. (2009) and Li et al. (2015) cannot be applied to Eq. (1) because there is another parameter $\beta$ and the condition on function *f* in Li et al. (2015), Erbe et al. (2009) does not satisfy the hypothesis $(H_5)$. In this paper, we will extend the results of Liu et al. (2012), Shi et al. (2016) to the case of $-1< p(t) \le 0$ and improve the results of Erbe et al. (2009), Li et al. (2015). By employing Riccati transformation, several new oscillation and asymptotic criteria are obtained under the assumptions that $(H_1)-(H_5)$. Throughout this paper, we suppose that all inequalities hold for sufficiently large *t*. Without loss of generality, we only consider the positive solutions of (1).

In what follows, let $D=\{(t, s):t_0\le s \le t\}\; \mathrm {and}\;D_0=\{(t, s):t_0\le s < t\}.$ We say a function $H=H(t,s)$ belongs to a function class *P*, if it satisfies

(i) $$H(t, t)=0, t\ge t_0; H(t,s)>0, (t, s)\in D_0;$$
(ii) *H* has partial derivatives $\partial H/\partial t$ and $\partial H{/}\partial s$ on $D_0$, such that
  $$\begin{aligned} \frac{\partial H(t, s)}{\partial t}=h_1(t, s)\sqrt{H(t, s)} \end{aligned}$$
  and
  $$\begin{aligned} \frac{\partial H(t, s)}{\partial s}=-h_2(t, s)\sqrt{H(t, s)}, \end{aligned}$$
  where $h_1$ and $h_2$ are nonnegative continuous functions on $D_0$.

## Main results

In this section, we discuss the Eq. (1) under the assumptions that $-1< p(t)\le 0$ and $0\le p(t)\le 1$, respectively.

### Oscillation of Eq. (1) when $-1<p(t)\le 0$

#### **Theorem 1**

*Assume that* $(H_2)-(H_5)$ *hold. If there exists a function* $\rho \in {\mathrm{C}}^1([t_0, \infty ), (0,\infty ))$ *such that, for any constant* $K>0$,

5 $$\begin{aligned} \int _{t_0}^\infty \left[ \rho (t)q(t)-\frac{\left( \rho ^{\prime }(t)\right) ^2}{4\rho ^2(t)\Phi (t)}\right] {\mathrm{d}}t =\infty , \end{aligned}$$

*where* $\Phi (t)=[\beta \sigma ^{\prime }(t)(\xi (\sigma (t)))^{\beta -1}]/[K^{(1-\beta /\alpha )}\rho (t)(r(\sigma (t)))^{1/\alpha }]$, *then all solutions of* Eq. (1) *are oscillatory or tend to zero as* $t\rightarrow \infty$.

#### *Proof*

Suppose *x* is a nonoscillatory solution of (1). Without loss of generality, there exists a $t_1\ge t_0$, such that $x(t)>0, x(\tau (t))>0,\;\text {and}\;x(\sigma (t))>0,$ for all $t\ge t_1$. From (1) and the hypothesis $(H_5)$, we get

6 $$\begin{aligned} \left( r|z^{\prime }|^{\alpha -1}z^{\prime }\right) ^{\prime }\le 0. \end{aligned}$$

Therefore, $r|z^{\prime }|^{\alpha -1}z^{\prime }$ is nonincreasing. We claim that $z^{\prime }>0$. Otherwise, if $z^{\prime }<0$, using the fact that $r|z^{\prime }|^{\alpha -1}z^{\prime }$ is nonincreasing, there exists a positive $k>0$, such that

$$\begin{aligned} -r(t)\left( -z^{\prime }(t)\right) ^{\alpha }\le -k<0. \end{aligned}$$

That is,

$$\begin{aligned} -z^{\prime }(t)\ge \frac{k^{\frac{1}{\alpha }}}{r^{\frac{1}{\alpha }}(t)}. \end{aligned}$$

Integrating the above inequality from $t_1$ to *t*, we get

$$\begin{aligned} z(t_1)-z(t) \ge k^{\frac{1}{\alpha }}\int _{t_1}^t r^{-\frac{1}{\alpha }}(s){\mathrm{d}}s. \end{aligned}$$

It follows from $(H_2)$ that

7 $$\begin{aligned} \lim _{t\rightarrow \infty }z(t)=-\infty . \end{aligned}$$

We consider the following two cases.

*Case 1* If *x* is unbounded, then there exists a sequence $\{t_m\}$, such that

$$\begin{aligned} \lim _{m\rightarrow \infty } x(t_m)=\infty , \end{aligned}$$

where $\{t_m\}$ satisfies $\lim _{m\rightarrow \infty }t_m=\infty$ and $x(t_m)=\max _{t_0\le s\le t_m}\{x(s)\}$. By the definition of $x(t_m)$ and $\tau (t)\le t$, we have

$$\begin{aligned} x(\tau (t_m))\le x(t_m). \end{aligned}$$

Then we get

$$\begin{aligned} z(t_m)=x(t_m)+p(t_m)x(\tau (t_m))\ge x(t_m)(1+p(t_k))>0, \end{aligned}$$

which contradicts (7).

*Case 2* If *x* is bounded, from the definition of *z* and $-1< p(t) \le 0$, *z* is also bounded, which also contradicts (7).

Hence, it is clear from the above discussion that $z^{\prime }>0$, and then $z>0$ or $z<0$. We consider each of two cases separately.

Suppose first that $z>0$. Considering the definition of *z* and $-1<p(t)\le 0$, we get

8 $$\begin{aligned} z(t)=x(t)+p(t)x(\tau (t))\le x(t). \end{aligned}$$

From $\sigma (t)\le t$ and the fact that $r(z^{\prime })^{\alpha }$ is nonincreasing, we obtain

9 $$\begin{aligned} \left( r(\sigma (t))\right) ^{\frac{1}{\alpha }}z^{\prime }(\sigma (t))\ge \left( r(t)\right) ^{\frac{1}{\alpha }}z^{\prime }(t) \end{aligned}$$

and there exist a positive constant *K* and a $t_2\ge t_1$, such that

10 $$\begin{aligned} r(t)\left( z^{\prime }(t)\right) ^{\alpha }\le K,\quad t\ge t_2. \end{aligned}$$

From the fact that $r(z^{\prime })^{\alpha }$ is nonincreasing, we get

11 $$z(t) = {} z(t_1)+\int _{t_1}^t \frac{\left( r(s)\left( z^{\prime }(s)\right) ^{\alpha }\right) ^{\frac{1}{\alpha }}}{r^{\frac{1}{\alpha }}(s)} {\mathrm{d}} s \ge r^{\frac{1}{\alpha }}(t)z^{\prime }(t)\int _{t_1}^t r^{-\frac{1}{\alpha }}(s){\mathrm{d}}s = \xi (t)r^{\frac{1}{\alpha }}(t)z^{\prime }(t),$$

where $\xi (t)=\int _{t_1}^t r^{-1/{\alpha }}(s) {\mathrm{d}}s$. Define a function $\omega$ by

$$\begin{aligned} \omega (t)=\rho (t)\frac{r(t)\left( z^{\prime }(t)\right) ^{\alpha }}{z^{\beta }(\sigma (t))}, \end{aligned}$$

then $\omega (t)>0$. Differentiating $\omega$, we get

$$\omega ^{\prime }(t) = \frac{\rho ^{\prime }(t)}{\rho (t)}\omega (t)+\rho (t)\frac{\left( r(t)z^{\prime }(t)\right) ^{\prime }}{z^{\beta }(\sigma (t))} - \beta \sigma ^{\prime }(t)\rho (t)\frac{r(t)\left( z^{\prime }(t)\right) ^{\alpha }z^{\beta -1}(\sigma (t))z^{\prime }(\sigma (t))}{z^{2\beta }(\sigma (t))}.$$

From (1) and (8), we conclude that

$$\begin{aligned} \omega ^{\prime }(t) \le -\rho (t)q(t)+\frac{\rho ^{\prime }(t)}{\rho (t)}\omega (t)-\beta \sigma ^{\prime }(t)\rho (t)\frac{r(t)\left( z^{\prime }(t)\right) ^{\alpha }}{z^{2\beta }(\sigma (t))} z^{\prime }(\sigma (t))z^{\beta -1}(\sigma (t)). \end{aligned}$$

Taking into account (11), the last inequality implies

12 $$\begin{aligned}&\omega ^{\prime }(t)\le - \rho (t)q(t)+\frac{\rho ^{\prime }(t)}{\rho (t)}\omega (t)-\beta \sigma ^{\prime }(t)(\xi (\sigma (t)))^{\beta -1}\nonumber \\&\quad \qquad \rho (t) \frac{r(t)\left( z^{\prime }(t)\right) ^{\alpha }}{z^{2\beta }(\sigma (t))} \left( r(\sigma (t))\right) ^{\frac{\beta -1}{\alpha }}\left( z^{\prime }(\sigma (t))\right) ^{\beta }. \end{aligned}$$

It follows from (9), (10), and (12) that

13 $$\begin{aligned} \omega ^{\prime }(t)&\le - \rho (t)q(t)+\frac{\rho ^{\prime }(t)}{\rho (t)}\omega (t)- \frac{\beta \sigma ^{\prime }(t)\left( \xi (\sigma (t))\right) ^{\beta -1}}{\left( r(\sigma (t))\right) ^{1/\alpha }} \rho (t)\frac{r(t)\left( z^{\prime }(t)\right) ^{\alpha }}{z^{2\beta }(\sigma (t))} \left( r(t)\right) ^{\frac{\beta }{\alpha }}\left( z^{\prime }(t)\right) ^{\beta }\\&=-\rho (t)q(t)+\frac{\rho ^{\prime }(t)}{\rho (t)}\omega (t)- \frac{\beta \sigma ^{\prime }(t)\left( \xi (\sigma (t))\right) ^{\beta -1}\left( r(t)\right) ^{\frac{\beta }{\alpha }}}{\left( r(\sigma (t))\right) ^{1/\alpha }\rho (t)r(t)} \omega ^2(t)\frac{1}{\left( z^{\prime }(t)\right) ^{\alpha -\beta }}\\&\le -\rho (t)q(t)+\frac{\rho ^{\prime }(t)}{\rho (t)}\omega (t)- \frac{\beta \sigma ^{\prime }(t)\left( \xi (\sigma (t))\right) ^{\beta -1}}{K^{1-\frac{\beta }{\alpha }}\left( r(\sigma (t))\right) ^{1/\alpha }\rho (t)} \omega ^2(t)\\&= -\rho (t)q(t)+\frac{\rho ^{\prime }(t)}{\rho (t)}\omega (t)-\Phi (t)\omega ^2(t)\\&\le -\rho (t)q(t)+\frac{(\rho ^{\prime }(t))^2}{4\rho ^2(t)\Phi (t)},\\ \end{aligned}$$

where $\Phi (t)=[\beta \sigma ^{\prime }(t)(\xi (\sigma (t)))^{\beta -1}]/[K^{(1-\beta /\alpha )}\rho (t)(r(\sigma (t)))^{1/\alpha }].$ Integrating (13) from $t_2$ to *t*, we get

$$\begin{aligned} 0<\omega (t)\le \omega (t_2)-\int ^t_{t_2}\left[ \rho (s)q(s)-\frac{\left( \rho ^{\prime }(s)\right) ^2}{4\rho ^2(s)\Phi (s)}\right] {\mathrm{d}}s, \end{aligned}$$

which contradicts (5).

If $z<0$, we claim that $\lim _ {t\rightarrow \infty }{x(t)}=0$. Using $z<0$ and $z^{\prime }>0$, we deduce that

$$\begin{aligned} \lim _{t\rightarrow \infty }z(t)=l\le 0, \end{aligned}$$

where *l* is a constant. That is, for all sufficiently large *t*, *z* is bounded. We can easily prove that *x* is also bounded. From the fact that *x* is bounded, we get

$$\begin{aligned} \limsup _{t\rightarrow \infty } x(t)=a, \end{aligned}$$

where *a* is a constant. We claim that $a=0$. Otherwise, if $a>0$, there exists a sequence $\{t_n\}$, such that $\lim _{n\rightarrow \infty }t_n=\infty$ and $\lim _{n\rightarrow \infty } x(t_n)=a.$ Letting

$$\begin{aligned} \varepsilon =\frac{a(1-p_0)}{2p_0}, \end{aligned}$$

for large enough *n*, we obtain

$$\begin{aligned} x(\tau (t_n))<a+\varepsilon . \end{aligned}$$

Then, from the definition of *z*(*t*) and $p(t)\ge -p_0$, we have

$$\begin{aligned} \lim _{n\rightarrow \infty }z(t_n)\ge \lim _{n\rightarrow \infty }x(t_n)-p_0(a+\varepsilon )=\frac{a(1-p_0)}{2}>0, \end{aligned}$$

which contradicts $z<0$. Thus $\limsup _{t\rightarrow \infty } x(t)=0.$ By $x>0$, we get

$$\begin{aligned} \lim _ {t\rightarrow \infty }{x(t)}=0. \end{aligned}$$

The proof is complete. $\square$

From Theorem 1, letting $\rho =1$, we get the following corollary.

#### **Corollary 1**

*Assume that* $(H_2)-(H_5)$ *hold. If*

$$\begin{aligned} \int _{t_0}^{\infty }q(t) {\mathrm{d}}t=\infty , \end{aligned}$$

*then all solutions of* Eq. (1) *are oscillatory or tend to zero as* $t\rightarrow \infty$.

#### **Theorem 2**

*Assume that* $(H_2)-(H_5)$ *hold. If there exist two functions* $H\in P$ *and* $\rho \in {\mathrm{C}}^1([t_0, \infty ), (0,+\infty ))$, *such that*

14 $$\begin{aligned} \limsup _{t\rightarrow \infty } \frac{1}{H(t,t_0)}\int _{t_0}^t\left[ H(t,s)\rho (s)q(s)-\frac{\left( \frac{\rho ^{\prime }(s)}{\rho (s)}H(t,s) -h_2(t,s)\sqrt{H(t,s)}\right) ^2}{4H(t,s)\Phi (s)}\right] {\mathrm{d}}s=\infty , \end{aligned}$$

*where* $\Phi$ *is as in* Theorem 1, *then the conclusion of* Theorem 1 *remains intact*.

#### *Proof*

Suppose that *x* is a nonoscillatory solution of (1). Proceeding as in the proof of Theorem 1, we get $z>0$ or $z<0$.

Firstly, we consider $z>0$. As the proof above (13) holds. That is

$$\begin{aligned} \omega ^{\prime }(t) \le -\rho (t)q(t)+\frac{\rho ^{\prime }(t)}{\rho (t)}\omega (t)-\Phi (t)\omega ^2(t). \end{aligned}$$

Multiplying this inequality by *H*(*t*, *s*) and integrating it from $t_2$ to *t*, we have

15 $$\begin{aligned} \int _{t_2}^t H(t,s)\omega ^{\prime }(s){\mathrm{d}}s \le \int _{t_2}^t H(t,s)\left[ -\rho (s)q(s)+ \frac{\rho ^{\prime }(s)}{\rho (s)}\omega (s)-\Phi (s)\omega ^2(s)\right] {\mathrm{d}}s. \end{aligned}$$

By the property of $\partial H(t, s)/\partial s=-h_2(t, s)\sqrt{H(t, s)}<0$, we conclude that

$$\begin{aligned}&\int _{t_2}^t\left[ H(t,s)\rho (s)q(s)-\frac{\left( \frac{\rho ^{\prime }(s)}{\rho (s)}H(t,s) -h_2(t,s)\sqrt{H(t,s)}\right) ^2}{4H(t,s)\Phi (s)}\right] {\mathrm{d}}s\nonumber \\&\quad \le H(t,t_2)\omega (t_2)\le H(t,t_0)\omega (t_2) . \end{aligned}$$

Adding $\int _{t_0}^{t_2}\left[H(t,s)\rho (s)q(s)- \frac{\left( \frac{\rho ^{\prime }(s)}{\rho (s)}H(t,s)-h_2(t,s)\sqrt{H(t,s)}\right) ^2}{4H(t,s)\Phi (s)}\right]{\mathrm{d}}s$ to the latter inequality and multiplying this inequality by $1/H(t,t_0)$, we get

16 $$\begin{aligned} &\frac{1}{H(t,t_0)}\left[ \int _{t_0}^{t_2}+\int _{t_2}^t\right] \left[ H(t,s)\rho (s)q(s)-\frac{\left( \frac{\rho ^{\prime }(s)}{\rho (s)}H(t,s)-h_2(t,s)\sqrt{H(t,s)}\right) ^2}{4H(t,s)\Phi (s)}\right] {\mathrm{d}}s\\&\quad \quad \le \frac{1}{H(t,t_0)}\int _{t_0}^{t_2}\left[ H(t,s)\rho (s)q(s)- \frac{\left( \frac{\rho ^{\prime }(s)}{\rho (s)}H(t,s)-h_2(t,s)\sqrt{H(t,s)}\right) ^2}{4H(t,s)\Phi (s)}\right] {\mathrm{d}}s + \omega (t_2).\\&\quad \quad \le \omega (t_2)+\int _{t_0}^{t_2}\left[ \frac{H(t,s)}{H(t,t_0)}\rho (s)q(s)- \frac{\left( \frac{\rho ^{\prime }(s)}{\rho (s)}\sqrt{H(t,s)}-h_2(t,s)\right) ^2}{4H(t,t_0)\Phi (s)}\right] {\mathrm{d}}s\\&\quad \quad \le \omega (t_2)+\int _{t_0}^{t_2}\rho (s)q(s) {\mathrm{d}}s.\\ \end{aligned}$$

Letting $t\rightarrow \infty$ in (16), we can get a contradict to (14).

If $z<0$, repeating the proof of Theorem 1, we have $\lim _ {t\rightarrow \infty }{x(t)}=0$. This completes the proof. $\square$

From Theorem 2, letting $H(t,s)=(t-s)^{\lambda } (\lambda >0)$ and $\rho (t)=1$, we may get the following corollary.

#### **Corollary 2**

*Assume that* $(H_2)-(H_5)$ *hold. If*

17 $$\begin{aligned} \limsup _{t\rightarrow \infty }\frac{1}{(t-t_0)^{\lambda }}\int _{t_0}^t (t-s)^{\lambda -2}\left[ (t-s)^2q(s)-\frac{\lambda ^2}{4\Phi (s)}\right] {\mathrm{d}}s=\infty , \end{aligned}$$

*then the conclusion of* Theorem 1 *remains intact*.

#### **Theorem 3**

*Assume that* $(H_2)-(H_5)$ *hold. If there exist three functions* $H\in P$, $\rho \in ([t_0, \infty ), (0,\infty ))$, *and* $A\in {\mathrm {C}}([t_0,\infty ), {\mathbb {R}})$, *such that*

18 $$\begin{aligned} \int _{t_0}^{\infty }A_{+}^2(t)\Phi (t){\mathrm{d}} t=\infty \end{aligned}$$

*and*

19 $$\begin{aligned}&\limsup _{t\rightarrow \infty }\frac{1}{H(t,T)}\nonumber \\&\quad \int _T^t\left[ h(t,s)\rho (s)q(s)- \frac{\theta \left( \frac{\rho ^{\prime }(s)}{\rho (s)}H(t,s)- h_2(t,s)\sqrt{H(t,s)}\right) ^2}{4H(t,s)\Phi (s)}\right] {\mathrm{d}}s\ge A(T), \end{aligned}$$

*for all* $T\ge t_0$ *and for some constant* $\theta >1$, *where* $A_{+}(t)=\max \{A(t),0\}$, $\Phi$ *is as in* Theorem 1, *and* *H* *satisfies*

20 $$\begin{aligned} 0<\inf _{s\ge t_0}\left\{ \limsup _{t\rightarrow \infty }\frac{H(t,s)}{H(t,t_0}\right\} \le \infty , \end{aligned}$$

*then every solution of* Eq. (1) *is oscillatory or tends to zero as* $t\rightarrow \infty$.

#### *Proof*

Suppose that *x* is a nonoscillatory solution of Eq. (1). Then, as in the proof of Theorem 1, $z>0$ or $z<0$.

We consider $z>0$ firstly. By virtue of Theorem 2, (15) holds. That is, for all $T\ge t_2\ge t_1,$

$$\begin{aligned} &\frac{1}{H(t,T)}\int _{T}^t H(t,s)\rho (s)q(s){\mathrm{d}}s\\&\quad \le \omega (T)+\frac{1}{H(t,T)} \int _{T}^t \frac{\theta \left( \frac{\rho ^{\prime }(s)}{\rho (s)}H(t,s) -h_2(t,s)\sqrt{H(t,s)}\right) ^2}{4\Phi (s)H(t,s)}{\mathrm{d}}s\\&\quad -\frac{1}{H(t,T)}\int _T^t\frac{\theta -1}{\theta }H(t,s)\Phi (s)\omega ^2(s){\mathrm{d}}s.\\ \end{aligned}$$

Thus,

21 $$\begin{aligned} &\frac{1}{H(t,T)}\int _T^t \left[ H(t,s)\rho (s)q(s)-\frac{\theta \left( \frac{\rho ^{\prime }(s)}{\rho (s)}H(t,s)- h_2(t,s)\sqrt{H(t,s)}\right) ^2}{4\Phi (s)H(t,s)}\right] {\mathrm{d}}s\\&\quad \quad \le \omega (T)-\frac{1}{H(t,T)}\int _T^t \frac{\theta -1}{\theta }H(t,s)\Phi (s)\omega ^2(s){\mathrm{d}}s.\\ \end{aligned}$$

Taking into account (19) and (21), we deduce that

$$\begin{aligned} A(T)+\liminf _{t\rightarrow \infty }\frac{1}{H(t,T)} \int _T^t \frac{\theta -1}{\theta }H(t,s)\Phi (s)\omega ^2(s){\mathrm{d}}s\le \omega (T), \end{aligned}$$

then

22 $$A(T)\le \omega (T),\quad \text {for all}\quad T\ge t_2,$$

and

23 $$\begin{aligned} \liminf _{t\rightarrow \infty }\frac{1}{H(t,T)} \int _T^t H(t,s)\Phi (s)\omega ^2(s){\mathrm{d}}s\le \frac{\theta }{\theta -1}\left( \omega (T)-A(T)\right) <\infty . \end{aligned}$$

Now we will prove that

$$\begin{aligned} \lim _{t\rightarrow \infty }\int _{T}^t\Phi (s)\omega ^2(s){\mathrm{d}}s<\infty . \end{aligned}$$

On the contrary, if

24 $$\begin{aligned} \lim _{t\rightarrow \infty }\int _{T}^t\Phi (s)\omega ^2(s){\mathrm{d}}s=\infty . \end{aligned}$$

By the condition (20), there exists a positive constant *c*, such that

25 $$\begin{aligned} \inf _{s\ge t_0}\left\{ \liminf _{t\rightarrow \infty }\frac{H(t,s)}{H(t,t_0)}\right\}>c>0. \end{aligned}$$

On the other hand, using (24), for arbitrary positive *M*, there exists a $t_3\ge T$, such that

$$\begin{aligned} \int _{T}^t \Phi (s)\omega ^2(s) {\mathrm{d}}s\ge \frac{M}{c}, \quad t\ge t_3. \end{aligned}$$

From the property (*ii*) of *H* and (25), we have

26 $$\begin{aligned} &\frac{1}{H(t,T)} \int _{T}^t H(t,s)\Phi (s)\omega ^2(s) {\mathrm{d}}s\\&\quad =\frac{1}{H(t,T)}\int _{T}^t\left[ -\frac{\partial H(t,s)}{\partial s}\right] \left[ \int _{T}^s \Phi (u)\omega ^2(u){\mathrm{d}}u\right] {\mathrm{d}}s\\&\quad \ge \frac{1}{H(t,T)}\int _{t_3}^t\left[ -\frac{\partial H(t,s)}{\partial s}\right] \left[ \int _{T}^s \Phi (u)\omega ^2(u){\mathrm{d}}u\right] {\mathrm{d}}s\\&\quad \ge \frac{M}{c}\frac{H(t,t_3)}{H(t,T)}\ge \frac{M}{c}\frac{H(t,t_3)}{H(t,t_0)}\ge M.\\ \end{aligned}$$

Taking account into the fact that *M* is arbitrary positive constant, (26) implies that

$$\begin{aligned} \lim _{t\rightarrow \infty }\frac{1}{H(t,T)} \int _{T}^t H(t,s)\Phi (s)\omega ^2(s) {\mathrm{d}}s=\infty , \end{aligned}$$

which contradicts (23). Thus,

$$\begin{aligned} \lim _{t\rightarrow \infty }\int _{T}^t\Phi (s)\omega ^2(s){\mathrm{d}}s<\infty . \end{aligned}$$

Using (22) and $\Phi (t)>0$, we have

$$\begin{aligned} \int _{T}^{\infty }A_{+}^2(t)\Phi (t){\mathrm{d}}t \le \int _{T}^{\infty }\omega ^2(t)\Phi (t){\mathrm{d}}t <\infty , \end{aligned}$$

which contradicts (18).

If $z<0$, repeating the proof in Theorem 1, we get $\lim _{t\rightarrow \infty }x(t)=0$. The proof is complete. $\square$

#### *Remark 1*

Theorem 1–3 and Corollaries 1 and 2 are the oscillation and asymptotic results of (1) under the assumption that $-1<p(t)\le 0$. However, the results in Liu et al. (2012), Shi et al. (2016) are established in the case where $0\le p(t)\le 1$. In the hypothesis $(H_5)$, there is another parameter $\beta$ and the condition on function *f* in Li et al. (2015), Erbe et al. (2009) does not satisfy $(H_5)$. Therefore, the results of Li et al. (2015), Erbe et al. (2009) can not apply to Eq. (1).

### Oscillation of Eq. (1) when $0\le p(t)\le 1$

#### **Theorem 4**

*Assume that* $(H_1)$ *and* $(H_3)-(H_5)$ *hold. If there exists a function* $\rho (t)\in {\mathrm{C}}^1([t_0,\infty ),(0,\infty ))$ such that for any positive number *M*,

27 $$\begin{aligned} \int _{t_0}^\infty \left[ \rho (t){\overline{p}}(t)- \frac{M^{1-\frac{\beta }{\alpha }}\left( \rho ^{\prime }(t)\right) ^2(r(\sigma (t)))^{\frac{1}{\alpha }}}{4\beta \sigma ^{\prime }(t)\rho (t)(\xi (t))^{\beta -1}}\right] {\mathrm{d}}t =\infty , \end{aligned}$$

*where* ${\overline{p}}(t)=q(t)(1-p(\sigma (t)))^\beta$ *and* $\xi (t)=\int _{t_1}^t r^{-1/{\alpha }}(s) {\mathrm{d}}s$, *then the* Eq. (1) *is oscillatory*.

#### *Proof*

See “Appendix”. $$\square$$

Letting $\rho (t)=1$, we can get the following result.

#### **Corollary 3**

*Assume that* $(H_1)$ *and* $(H_3)-(H_5)$ *hold. If*

$$\begin{aligned} \int _{t_0}^\infty q(t)(1-p(\sigma (t)))^\beta {\mathrm{d}}t =\infty , \end{aligned}$$

*then the* Eq. (1) *is oscillatory.*

#### *Example 1*

Consider the second-order nonlinear neutral delay differential equation

28 $$\begin{aligned} \left( \left| \left( x(t)+\left( 1-t^{-\frac{1}{2}}\right) x(\tau (t))\right) ^{\prime }\right| ^{\alpha -1}\left[ x(t)+\left( 1-t^{-\frac{1}{2}}\right) x(\tau (t))\right] ^{\prime }\right) ^{\prime }+\frac{\gamma }{t^2}|x(t)|x(t)=0, \end{aligned}$$

where $\beta =2, \alpha \ge 2, r(t)=1, p(t)=1-t^{-\frac{1}{2}}, \sigma (t)=t,\; \text {and} \;q(t)=\frac{\gamma }{t^2}$, where $\gamma$ is a positive constant.

Letting $\rho (t)=t^2$, we have

$$\begin{aligned} \xi (t)=\int _{t_1}^t r^{-1/{\alpha }}(s) {\mathrm{d}}s=\int _{t_1}^t 1 {\mathrm{d}}s=t-t_1 \end{aligned}$$

and

$$\begin{aligned} &\int _T^t\left[ \rho (t){\overline{p}}(t)- \frac{\left( \rho ^{\prime }(t)\right) ^2(r(\sigma (t)))^{\frac{1}{\alpha }})}{4\beta \sigma ^{\prime }(t)M^{1-\frac{\beta }{\alpha }} \rho (t)(\xi (t))^{\beta -1}}\right] {\mathrm{d}}s= \int _T^t \left[ \frac{\gamma }{s}-\frac{1}{2K^{1-\frac{2}{\alpha }}(s-t_1)}\right] {\mathrm{d}} s\\&\quad =\gamma \ln t-\frac{1}{2M^{1-\frac{2}{\alpha }}}\ln (t-t_1)-\gamma \ln T+\frac{1}{2M^{1-\frac{2}{\alpha }}}\ln (T-t_1). \end{aligned}$$

Therefore, if $\gamma > \frac{1}{2M^{1-(2/{\alpha })}}$, then

$$\begin{aligned} \int _T^{\infty }\left[ \rho (t){\overline{p}}(t)- \frac{\left( \rho ^{\prime }(t)\right) ^2(r(\sigma (t)))^{\frac{1}{\alpha }})}{4\beta \sigma ^{\prime }(t)M^{1-\frac{\beta }{\alpha }} \rho (t)(\xi (t))^{\beta -1}}\right] {\mathrm{d}}s=\infty . \end{aligned}$$

It follows from Theorem 4 that all solutions of (28) are oscillatory if $\gamma > \frac{1}{2M^{1-(2/{\alpha })}}$. However, the Eq. (28) is oscillatory when $\gamma > \frac{1}{M^{1-(2/{\alpha })}}$ from Theorem 1 in Liu et al. (2012). That is, if $\frac{1}{2M^{1-(2/{\alpha })}}<\gamma \le \frac{1}{M^{1-(2/{\alpha })}}$, then Theorem 1 in Liu et al. (2012) can not apply to (28).

#### *Remark 2*

From Theorem 4, the Eq. (28) is oscillatory if $\gamma > \frac{1}{2M^{1-(2/{\alpha })}}$. However, from Theorem 1 in Liu et al. (2012), when $\gamma > \frac{1}{M^{1-(2/{\alpha })}}$, the Eq. (28) is oscillation. Thus, Theorem 4 improves Theorem 1 in Liu et al. (2012) in the case where $0\le p(t)\le 1$.

## Examples

In this section, we will present two examples to illustrate the main results.

### *Example 2*

Consider the second-order nonlinear neutral delay differential equation

29 $$\begin{aligned} \left( \left| \left( x(t)-\frac{1}{2} x(t-2)\right) ^{\prime }\right| ^{\alpha -1}\left[ x(t)-\frac{1}{2} x(t-2)\right] ^{\prime }\right) ^{\prime }+2\left( \frac{e^2}{2}-1\right) ^2|x(t)|^{\beta -1}x(t)=0, \end{aligned}$$

where $$r(t)=1, p(t)=-1/2, \tau (t)=t-2, \sigma (t)=t,\; \text {and} \;q(t)=2\left( \frac{e^2}{2}-1\right) ^2$$.

We see that

$$\begin{aligned} \int _T^{\infty } q(s){\mathrm{d}}s=\infty . \end{aligned}$$

It follows from Corollary 1 that all solutions of (29) are oscillatory or converge to zero. Letting $\alpha =\beta =2$, we can certify that $x(t)=e^{-t}$ is an asymptotic solution of (29).

### *Example 3*

Consider the second-order nonlinear neutral delay differential equation

30 $$\begin{aligned} \left( \left| (x(t)-\lambda _0 x(\tau (t)))^{\prime }\right| ^{\alpha -1}[x(t)-\lambda _0 x(\tau (t))]\right) ^{\prime }+\frac{\gamma }{t^2} |x(\sigma _0t)|^{\beta -1}x(\sigma _0t)=0, \end{aligned}$$

where $$\alpha >\beta \ge 1$$, $$0<\lambda _0<1, 0<\sigma _0<1$$, $$r(t)=1, p(t)=\lambda _0, \tau (t)\le t, q(t)=\frac{\gamma }{t^2},$$ and $$\sigma (t)=\sigma _0t$$.

Letting $\rho (t)=\frac{\gamma }{2} \frac{\beta \sigma _0\left( t-\frac{t_1}{\sigma _0}\right) ^{\beta -1}}{K^{1-\frac{\beta }{\alpha }}}$ and $H(t,s)=(t-s)^2$, then

$$\begin{aligned} \xi (t)&= {} \int _{t_1}^t r^{-\frac{1}{\alpha }}(s) {\mathrm{d}}s=t-t_1,\\ \Phi (t)&= {} \frac{\beta \sigma ^{\prime }(t)(\xi (\sigma (t)))^{\beta -1}}{K^{1-\frac{\beta }{\alpha }}\rho (t)(r(\sigma (t)))^{\frac{1}{\alpha }}} =\frac{\beta \sigma _0(t-\frac{t_1}{\sigma _0})^{\beta -1}}{K^{1-\frac{\beta }{\alpha }}\rho (t)}=\frac{2}{\gamma } , \end{aligned}$$

and

$$\begin{aligned} \limsup _{t\rightarrow \infty }\frac{1}{(t-s)^2}\int _{t_0}^t\left[ (t-s)^2q(s)-\frac{1}{\Phi (s)}\right] {\mathrm{d}}s=\limsup _{t\rightarrow \infty }\frac{1}{(t-s)^2}\int _{t_0}^t\left[ \gamma (\frac{1}{2}-\frac{2t}{s}+\frac{t^2}{s^2})\right] {\mathrm{d}}s=\infty . \end{aligned}$$

That is, (17) holds. It follows from Corollary 2 that all solutions of (30) are oscillatory or converge to zero.

### *Remark 3*

If $\alpha >\beta$, the results of Erbe et al. (2009) and Li et al. (2015) can not apply to (29) and (30). The results of Liu et al. (2012) and Shi et al. (2016) also can not apply to (29) and (30) because $-1< p(t)\le 0$ which does not satisfy the assumptions in Liu et al. (2012), Shi et al. (2016).

## Conclusion

In this paper, we consider the oscillation of a class of second-order differential equations with positive and nonpositive neutral coefficients. It is difficult to study the nonpositive neutral coefficients equations because the sign of *z* is not explicit. Using Riccati transformation, some oscillation and asymptotic criteria are obtained under the assumptions that $(H_1)-(H_5)$. In Liu et al. (2012), Shi et al. (2016), the results were established for (1) in the case when $0\le p(t)\le 1$ or $p(t)>1$. This paper states some oscillation and asymptotic criteria for (1) in the case where $-1<p(t)<0$ and $0\le p(t)\le 1$. Erbe et al. (2009), Li et al. (2015) assume that $\alpha =\beta$, however, in this paper $\alpha \ne \beta$ is allowed. We give some examples to illustrate our results. There are two interesting questions for future study:

1. Could we obtain oscillation criteria for Eq. (1) when $-\infty < p(t)\le 1\, \text{or} -\infty<p(t)\le-1$?
2. Could we obtain some sufficient conditions which ensure that all solutions of (1) are oscillatory?

## Appendix

In this section, we give the proof of Theorem 4.

### *Proof*

Assume that *x* is a nonoscillatory solution of the Eq. (1). Without loss of generality, there exists a $t_1\ge t_0$, such that $x(t)>0, x(\tau (t))>0,\; \text {and}\; x(\sigma (t))>0$, for all $t\ge t_1$. Then, by the definition of *z*, we know

31 $$\begin{aligned} z(t)=x(t)+p(t)x(\tau (t))\ge x(t)\quad \text {and}\quad z>0. \end{aligned}$$

From (1) and the assumption $(H_5)$, we get

32 $$\begin{aligned} \left( r|z^{\prime }|^{\alpha -1}z^{\prime }\right) ^{\prime }\le 0. \end{aligned}$$

So, $r|z^{\prime }|^{\alpha -1}z^{\prime }$ is a nonincreasing function. We claim that $z^{\prime }>0$. Otherwise, if $z^{\prime }<0$, using the fact that $r|z^{\prime }|^{\alpha -1}z^{\prime }$ is a nonincreasing, there exists a positive $c>0$, such that

$$\begin{aligned} -r\left( -z^{\prime }\right) ^{\alpha }\le -c<0. \end{aligned}$$

Then, we have

$$\begin{aligned} -z^{\prime }(t)\ge \left( \frac{c}{r(t)}\right) ^{\frac{1}{\alpha }}. \end{aligned}$$

Integrating the above inequality from $t_1$ to *t*, we obtain that

$$\begin{aligned} z(t_1)-z(t)\ge c^{\frac{1}{\alpha }}\int _{t_1}^t \left( \frac{1}{r(t)}\right) ^{\frac{1}{\alpha }} {\mathrm{d}}s \end{aligned}$$

Letting $t\rightarrow \infty$, from $(H_1)$, we have

$$\begin{aligned} \lim _{t\rightarrow \infty }z(t)=-\infty , \end{aligned}$$

which contradicts $z>0$. Thus, $z^{\prime }>0$.

From (1), (31), and the fact that *z* is increasing, we conclude that

33 $$\begin{aligned} \left( r(t)\left( z^{\prime }(t)\right) ^\alpha \right) ^{\prime }+q(t)z^\beta (\sigma (t))\left( 1-p(\sigma (t))\right) ^\beta \le 0 , \end{aligned}$$

Using the fact that $r(z^{\prime })^{\alpha }$ is nonincreasing, (11) holds and there exists a positive constant *M* and $t_2\ge t_1$, such that

34 $$\begin{aligned} r(t)\left( z^{\prime }(t)\right) ^{\alpha }\le M, \quad t\ge t_2 , \end{aligned}$$

and form $\sigma (t)\le t$, we get

35 $$\begin{aligned} r(t)\left( z^{\prime }(t)\right) ^{\alpha }\le r(\sigma (t))\left( z^{\prime }(\sigma (t))\right) ^{\alpha }. \end{aligned}$$

Define a function $\omega$ by

$$\begin{aligned} \omega (t)=\rho (t)\frac{r(t)\left( z^{\prime }(t)\right) ^{\alpha }}{z^{\beta }(\sigma (t))}, \end{aligned}$$

then $\omega (t)>0$. Differentiating $\omega$, we get

$$\begin{aligned} \omega ^{\prime }(t)=\frac{\rho ^{\prime }(t)}{\rho (t)}\omega (t)+\rho (t)\frac{\left( r(t)z^{\prime }(t)\right) ^{\prime }}{z^{\beta }(\sigma (t))}- \beta \sigma ^{\prime }(t)\rho (t)\frac{r(t)\left( z^{\prime }(t)\right) ^{\alpha }z^{\beta -1}(\sigma (t))z^{\prime }(\sigma (t))}{z^{2\beta }(\sigma (t))}. \end{aligned}$$

From (33), we conclude that

$$\begin{aligned} \omega ^{\prime }(t) \le -\rho (t)q(t){\overline{p}}(t)+\frac{\rho ^{\prime }(t)}{\rho (t)}\omega (t)-\beta \sigma ^{\prime }(t)\rho (t)\frac{r(t)\left( z^{\prime }(t)\right) ^{\alpha }}{z^{2\beta }(\sigma (t))} z^{\prime }(\sigma (t))z^{\beta -1}(\sigma (t)), \end{aligned}$$

where ${\overline{p}}(t)=(1-p(\sigma (t)))^\beta$. Taking into account (11) and $\alpha \ge \beta \ge 1$, the last inequality implies

36 $$\begin{aligned}&\omega ^{\prime }(t)\le - \rho (t)q(t){\overline{p}}(t)+\frac{\rho ^{\prime }(t)}{\rho (t)}\omega (t)-\beta \sigma ^{\prime }(t)(\xi (\sigma (t)))^{\beta -1}\nonumber \\&\quad \rho (t) \frac{r(t)\left( z^{\prime }(t)\right) ^{\alpha }}{z^{2\beta }(\sigma (t))} \left( r(\sigma (t))\right) ^{\frac{\beta -1}{\alpha }}\left( z^{\prime }(\sigma (t))\right) ^{\beta }. \end{aligned}$$

It follows from (34) to (36) that

37 $$\begin{aligned} \omega ^{\prime }(t)&\le - \rho (t)q(t){\overline{p}}(t)+\frac{\rho ^{\prime }(t)}{\rho (t)}\omega (t)- \frac{\beta \sigma ^{\prime }(t)\left( \xi (\sigma (t))\right) ^{\beta -1}}{\left( r(\sigma (t))\right) ^{1/\alpha }} \rho (t)\frac{r(t)\left( z^{\prime }(t)\right) ^{\alpha }}{z^{2\beta }(\sigma (t))} \left( r(t)\right) ^{\frac{\beta }{\alpha }}\left( z^{\prime }(t)\right) ^{\beta }\\&=-\rho (t)q(t){\overline{p}}(t)+\frac{\rho ^{\prime }(t)}{\rho (t)}\omega (t)- \frac{\beta \sigma ^{\prime }(t)\left( \xi (\sigma (t))\right) ^{\beta -1}\left( r(t)\right) ^{\frac{\beta }{\alpha }}}{\left( r(\sigma (t))\right) ^{1/\alpha }\rho (t)r(t)} \omega ^2(t)\frac{1}{\left( z^{\prime }(t)\right) ^{\alpha -\beta }}\\&\le -\rho (t)q(t){\overline{p}}(t)+\frac{\rho ^{\prime }(t)}{\rho (t)}\omega (t)- \frac{\beta \sigma ^{\prime }(t)\left( \xi (\sigma (t))\right) ^{\beta -1}}{M^{1-\frac{\beta }{\alpha }}\left( r(\sigma (t))\right) ^{1/\alpha }\rho (t)} \omega ^2(t)\\&\le -\rho (t)q(t){\overline{p}}(t)+\frac{ M^{1-\frac{\beta }{\alpha }}(\rho ^{\prime }(t))^2}{4\beta \sigma ^{\prime }(s)\rho (t)(\xi (\sigma (t)))^{\beta -1} }.\\ \end{aligned}$$

Integrating (37) from $t_2$ to *t*, we get

$$\begin{aligned} 0<\omega (t)\le \omega (t_2)-\int ^t_{t_2}\left[ \rho (t)q(t){\overline{p}}(t)-\frac{ M^{1-\frac{\beta }{\alpha }}\left( \rho ^{\prime }(t)\right) ^2}{4\beta \sigma ^{\prime }(s)\rho (t)(\xi (\sigma (t)))^{\beta -1} }\right] {\mathrm{d}}s, \end{aligned}$$

which contradicts (27). This completes the proof. $\square$
